# Genetic analysis of chorionic villus tissues in early missed abortions

**DOI:** 10.1038/s41598-023-48358-0

**Published:** 2023-12-11

**Authors:** Huili Xue, Qun Guo, Aili Yu, Min Lin, Xuemei Chen, Liangpu Xu

**Affiliations:** 1 Medical Genetic Diagnosis and Therapy Center, Fujian Key Laboratory for Prenatal Diagnosis and Birth Defect, Fujian Maternity and Child Health Hospital College of Clinical Medicine for Obstetrics & Gynecology and Pediatrics, Fujian Medical University, Gulou District, No. 18 Daoshan Road, Fuzhou, 350001 Fujian China; 2 Reproductive Medicine Center, Fujian Maternity and Child Health Hospital College of Clinical Medicine for Obstetrics & Gynecology and Pediatrics, Fujian Medical University, Gulou District, No. 18 Daoshan Road, Fuzhou, 350001 Fujian China

**Keywords:** Genetics, Medical research

## Abstract

Chromosomal abnormalities are the most common etiology of early spontaneous miscarriage. However, traditional karyotyping of chorionic villus samples (CVSs) is limited by cell culture and its low resolution. The objective of our study was to investigate the efficiency of molecular karyotyping technology for genetic diagnosis of early missed abortion tissues. Chromosome analysis of 1191 abortion CVSs in early pregnancy was conducted from August 2016 to June 2021; 463 cases were conducted via copy-number variations sequencing (CNV-seq)/quantitative fluorescent-polymerase chain reaction (QF-PCR) and 728 cases were conducted using SNP array. Clinically significant CNVs of CVSs were identified to clarify the cause of miscarriage and to guide the couples’ subsequent pregnancies. Among these, 31 cases with significant maternal cell contamination were removed from the study. Among the remaining 1160 samples, 751 cases (64.7%) with genetic abnormalities were identified, of which, 531 (45.8%) were single aneuploidies, 31 (2.7%) were multiple aneuploidies, 50 (4.3%) were polyploidies, 54 (4.7%) were partial aneuploidies, 77 (6.6%) had submicroscopic CNVs (including 25 with clinically significant CNVs and 52 had variants of uncertain significance), and 8 cases (0.7%) were uniparental disomies. Our study suggests that both SNP array and CNV-seq/QF-PCR are reliable, robust, and high-resolution technologies for genetic diagnosis of miscarriage.

## Introduction

Spontaneous miscarriage in early pregnancy occurs in ~ 10–15% of all clinically recognized pregnancies^1^. Embryonic chromosomal abnormalities, reproductive immune disorders, anatomic abnormalities of the uterus, thrombophilias, endocrine diseases, infectious disorders, sperm quality, and environmental issues are all related to miscarriage^2^. Among these, embryonic chromosomal anomalies are the most common etiology of miscarriages, representing more than half of early spontaneous abortions^3^.

Traditional cytogenetic analyses of chorionic villus samples (CVSs) involve cell culture and karyotyping analysis, which is hindered by the low success rates of cell culture as well as low accuracy and resolution, as selective overgrowth of maternally derived cells^4^. The development of molecular karyotyping techniques, such as chromosomal microarray analysis (CMA) and copy number variation (CNV) sequencing (CNV-seq) have great advantages, including no cell culture required, as well as having high throughput analysis and resolution^5^. Both high-resolution molecular technologies have increasingly been applied for analysis of miscarriage specimens^6,7^. CNV-seq is a high-resolution and low-cost sequencing technology. The combination of CNV-seq and quantitative fluorescent-polymerase chain reaction (QF-PCR) can identify chromosome aneuploidies, polyploidies, and CNVs^8,9^.

Embryonic chromosomal numerical abnormalities accounted for up to 90% of the causes of early miscarriage^10^. CNVs are genomic copy number changes with sizes ranging from above 50 bp to Mb^11^. It is well known that segmental deletion and/or duplication can cause miscarriage. Additionally, submicroscopic pathogenic CNVs (pCNVs) are the second-highest aberrations identified in CVSs^12^. However, specific data regarding the association between submicroscopic CNVs and spontaneous miscarriage is still limited. Here, we investigated the feasibility and accuracy of two approaches, microarray analysis and the combination of CNV-seq and QF-PCR, for molecular karyotyping analysis of 1191 CVSs from early missed abortions in a tertiary referral center.

## Methods

### Study samples and genomic DNA extraction

Among 1191 patients agreed to undergo chromosome analysis of their aborted CVSs collected via hysterosuction. All the women with missed abortions underwent a standardized diagnostic workup in our center. In short, the villi tissues were washed with 0.9% sodium chloride solution under sterile condition, and the hemagglutination and decidual tissues of the mother were separated. Then, high quality villi were chosen for genomic DNA extraction. DNA was extracted using a QIAamp DNA Mini Kit (Qiagen, Hilden, Germany) according to the manufacture’s instructions, and the purity and concentration of genomic DNA were further determined by NanoDrop 2000 Ultramicro Spectrophotometer. Peripheral blood from both the pregnant couple were also collected to rule out maternal cell contamination (MCC) and to contribute to report interpretation if necessary. The participants’ age ranged from 19 to 48 years old, with an average age of 31.2 years old. The gestational age of all the miscarriages ranged from 5 to 12 weeks, with an average gestational age of 8.5 weeks. The participants were divided into subgroups based on maternal age, the number of previous miscarriages, and gestational weeks at miscarriage (Table 1). Informed consent was obtained from all participants. The study was conducted under the guidance of the Declaration of Helsinki and approved by the Ethics Committee of the Fujian Maternal and Child Health Hospital (No. 2020KYLLD01051).

**Table 1:** Demographic characteristics of 1160 cases with early missed abortion by molecular karyotyping analysis.

Variants	N	%
Maternal age (years)		
≤ 29	386	33.3
30–34	510	44.0
35–39	220	19.0
≥ 40	44	3.7
Previous abortion at ≤ 12 weeks		
0	352	30.3
1	194	16.8
2	462	39.8
≥ 3	152	13.1
Gestational weeks at miscarriage		
< 7 weeks	158	13.6
7–8^+6^ weeks	453	39.1
9–10^+6^ weeks	387	33.4
11–12 weeks	162	14.0

### QF-PCR

After genomic DNA extraction, QF-PCR was carried out to exclude MCC as reported previously^13^, using 15 polymorphic short tandem repeat (STR) markers, including three markers (D21S1435, D21S11 and D21S1411) for chromosome 21, four markers (D18S1002, D18S391, D18S535 and D18S386) for chromosome 18, four markers (D13S628, D13S742, D13S634 and D13S305) for chromosome 13, and four markers (DXS981, DXS6809, X22 and AMXY) for sex chromosomes. PCR products were electrophoresed on an ABI 3500 DX genetic analyzer and results were analyzed by ABI GeneMapper software (Applied Biosystems, Foster City, CA, USA).

### CNV sequencing/QF-PCR

According to its detection principle, CNV-seq alone cannot recognize SNP site to exclude MCC. Thus, for CNV-Seq technology, samples should be first investigated using QF-PCR assays or STR test to exclude significant MCC and triploidy.

CVSs samples were subjected for CNV-seq after exclusion of MCC. A total of 449 cases of chromosomal analysis was ultimately conducted by CNV sequencing (CNV-seq) due to 14 cases with significant MCC. Briefly, 10 mg of CVSs were selected under sterile conditions, DNA was extracted by Qiagen kit for later use, DNA library was constructed using dsDNA fragmentase to produce smaller fragments, end repair, A-tail and then ligated with barcoded sequencing adaptors. Single-end sequencing was performed on the the NextSeq CN500 platform to produce to produce approximately 5 million reads per sample. The number of reads after sample quality control is more than 8 M Sequence depth is ~ 1x. Each sequencing tag was matched to the corresponding chromosome by comparison software, and then standardized Z-value analysis was performed to determine chromosome copy number variation. On average, approximately 2.8 ~ 3.2 million reads were uniquely mapped for data analysis. The uniquely mapped reads were aligned to the human genomic reference sequences (hg19) using the Burrows-Wheeler algorithm and allocated to 20-kilobase (kb) bin on each chromosome^14^. To eliminate the influence of GC bias between different samples, GC correction was performed via LOESS regression, intrarun normalization, and linear model regression^15^, and CNVs were identified from 24 chromosome copy number plots. The pathogenicity of detected CNVs was assessed following American College of Medical Genetics (ACMG) guidelines. Genomic variant databases including DGV, DECIPHER , OMIM, PubMed and UCSC were used as a reference source of CNVs^16^.

The CNV-seq data analysis processes include (1) Quality control of raw sequencing data (fastq), removing adapters/low-quality bases, resulting in filtered fastq data. (2) Alignment of filtered fastq data to the human reference genome (hg19) using alignment software like BWA. (3) Sorting and deduplication of the alignment results. (4) Binning the sorted results using genomic intervals to obtain read-depth profiles (sample-wise median normalization is applied, where the reads count in each bin is divided by the median reads count of the bins in autosomes. This step aims to mitigate biases introduced by different sequencing depths. (5) GC correction of the read-depth profiles. (6) CNV calling based on the GC-corrected read-depth profiles to detect CNV variations. (7) Analysis and interpretation of CNVs according to ACMG/ClinGen guidelines, including classification based on their clinical significance.
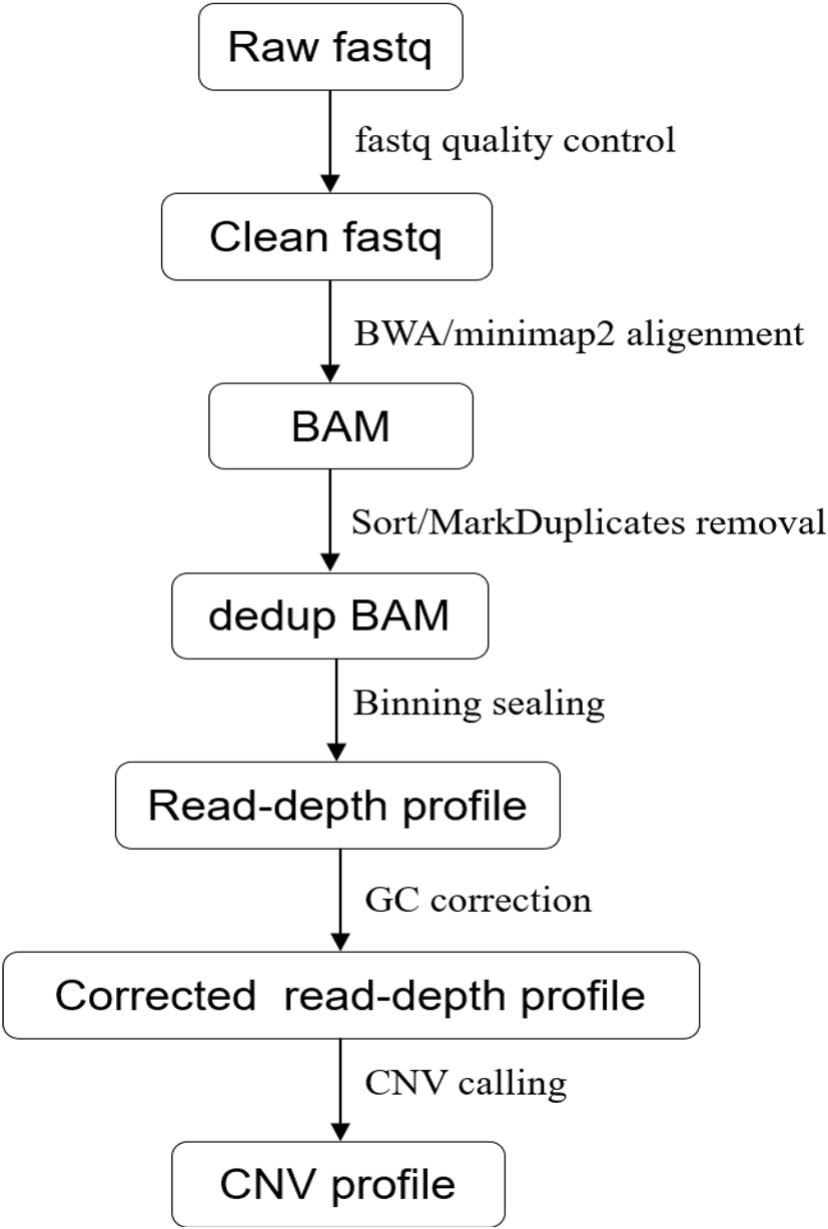


### SNP array

Due to the platform transition in our laboratory during the study period, totally, 711 cases of chromosomal CNVs (above 0.1 Mb) analysis were ultimately detected by SNP array due to 17 cases with significant MCC. The experimental process of SNP array was carried out using Affymetrix CytoScan 750 K array (Affymetrix Inc., Santa Clara, CA) as previously described^17^. The data was analyzed using Affymetrix Chromosome Analysis Suite Software (version 3.1.0.15). Parental microarray testing was carried out to determine its origin. Pathogenicity of CNVs is classified according to ACMG guideline^16^. Only P/LP CNVs and VOUS are reported in this study. Large CNVs (≥ 10 Mb) were classified as partial aneuploidies, and submicroscopic CNVs (< 10 Mb) were defined as microdeletions and microduplications. Large CNVs detected in three or more cases were defined as recurrent large CNVs, while submicroscopic CNVs found in two or more cases were considered as recurrent submicroscopic CNVs.

### Parental conventional karyotyping analysis

Peripheral blood lymphocyte was isolated from the abortion couples when the chromosomal aberrations of CVSs suggesting parental balanced rearrangement, then were cultured and harvested after phytohemagglutinin stimulation for 72 h. Metaphase chromosomes were prepared following standard cytogenetic protocols.

Karyotypes were scanned on Leica GSL120. At least 20 metaphases were counted, and five metaphases were analyzed. The naming of abnormal karyotypes was based on ISCN 2020.

### Fluorescence in situ hybridization

Fluorescence in situ hybridization (FISH) using telomere probes (Vysis, IL) was performed to identify occult reciprocal balanced translocations carriers as reported previously^18^. After 72 h stimulation with phytohemagglutinin, the lymphocytes were cultured and harvested after stimulation using phytohemagglutinin for 72 h, and metaphase chromosomes were fixed on slides. After degeneration for 5 min at 78 °C, the fluorescent labeled probes were hybridized with the chromosomes on the slide at 37 °C for 16 h. The slides were washed in 2 × SSC (Sigma, USA) and stained with DAPI (Vysis, IL). The signal was observed under a fluorescence microscope (Leica, Germany).

### Statistical analysis

SPSS software version 19.0 (SPSS, Inc., Chicago, IL) was used for statistical analysis. Measurement data were expressed as mean ± standard deviation, statistical comparisons were performed using χ^2^ test (Fisher’s exact test), and *p* < 0.05 was considered statistically significant.

### Ethics approval and consent to participate

The study complied with the principles set forth in the Declaration of Helsinki. It was approved by the Institutional Review Board of Fujian Maternal and Child Health Hospital. Written informed consent was obtained from each patient.

## Results

### Baseline characteristics of the participants and chromosomal abnormalities of CVSs detected by molecular karyotyping technologies

**Figure 1:**
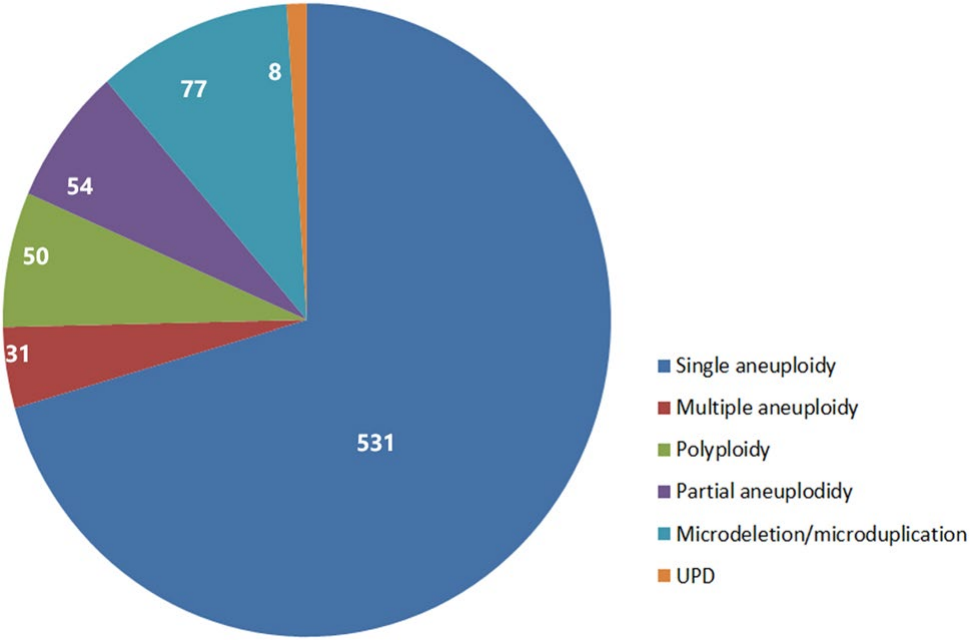
The constitution of chromosome abnormalities in CVSs. Overall, 751 samples (64.7%) with abnormal chromosomes were identified among 1160 CVSs, including 531 single aneuploidies, 31multiple aneuploidies, 50 polyploidies, 54 partial aneuplodidy, 77 microdeletion/microduplication, and 8 UPD. UPD—uniparental disomy.

Initially, we analyzed a total of 1191 cases by CNV-seq/QF-PCR or SNP array August 2016 to June 2021. Among these, 31 cases with serious MCC were removed. The remaining 1160 cases with no MCC were available for further molecular karyotyping analysis (Table 1), of which 449 cases were tested via CNV-seq/QF-PCR, and 711 cases were tested using SNP array. All 1160 CVSs were successfully analyzed. Thus, the detection success rate was 100% (1160/1160). As detection coverage was theoretically consistent except uniparental disomy (UPD) based on SNP array and CNV-seq/QF-PCR, we counted the results except UPD detected by the two methods together. Overall, 751 cases (64.7%) with genetic abnormalities were identified, of which, 531 (45.8%) were single aneuploidies, 31 (2.7%) were multiple aneuploidies, 50 (4.3%) were polyploidies, 54 (4.7%) were partial aneuploidies, 77 (6.6%) had CNVs (including 52 (4.5%) had VOUS), and 8 cases (0.7%) were UPD (Fig. 1). In addition, a total of 213 female samples and 196 male samples were found in the normal samples, with a female-to-male ratio of 1.09.

**Figure 2:**
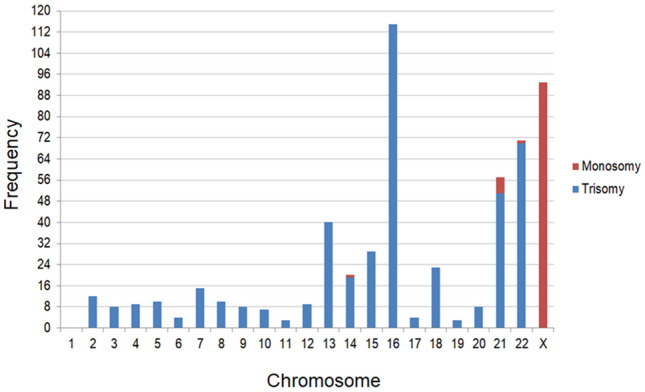
The frequency of each chromosomal aneuploidy.

Aneuploidy was the most common abnormal result, with 531 (45.8%) single aneuploidies and 31 (2.7%) multiple aneuploidies being identified (Fig. 2). Trisomy accounted for most single aneuploidies (430/531, 81.0%). Of the single types of trisomy, trisomy 16 was the most common (115/430, 26.7%), followed by trisomy 22 (70/430, 16.3%), trisomy 21 (51/430, 11.9%), and trisomy 13, 15, and 18. Monosomy was identified in 19.0% (101/531) of the cases with single aneuploidy; of which, monosomy X accounted for 92.1% (93/101) of these cases, while the remaining autosomal monosomy comprised the remaining 8.9% (9/101), with six cases of monosomy 21, one of monosomy 14, and one of monosomy 22.

Polyploidy was found in 50 (4.3%) cases: triploidy in 45 (3.9%) cases and hypotriploidy in 5 (0.4%) cases (Table 2).

**Table 2:** Types and frequencies of chromosomal abnormalities detected in 1160 CVSs.

Chromosomal abnormality	CNV-seq/QF-PCR (n = 449)		SNP array (n = 711)		Total (n = 1160 )	
	n	Frequency (%)	n	Frequency (%)	n Frequency (%)	
Single aneuploidy	**201**	**44.8**	**330**	**46.4**	**531**	**45.8**
Autosomal trisomy	127	28.3	253	35.6	380	32.8
Autosomal monosomy	4	0.9	4	0.6	8	0.7
Monosomy X	50	11.1	43	6.0	93	8.0
Sex chromosome trisomy	0	0.0	4	0.6	4	0.3
Mosaicism trisomy	20	4.5	26	3.7	46	4.0
Multiple aneuploidy	**9**	**2.0**	**22**	**3.1**	**31**	**2.7**
Double aneuploidy	9	2.0	21	3.0	30	2.6
Triple aneuploidy	0	0.0	1	0.1	1	0.0
Quadruple aneuploidy	0	0.0	0	0.0	0	0.0
Polyploidy	**28**	**6.2**	**22**	**3.1**	**50**	**4.3**
Triploidy	25	5.6	20	2.8	45	3.9
Hypotriploidy	3	0.7	2	0.3	5	0.4
Partial aneuplodidy (large CNVs ≥ 10 Mb)	**31**	**6.9**	**23**	**3.1**	**54**	**4.7**
Terminal deletion/duplication	10	2.2	8	1.1	18	1.6
Terminal deletion + terminal duplication involved in two chromosomes	9	2.0	8	1.1	17	1.5
Terminal deletion/duplication + interstitial deletion/duplication involve in one chromosome	1	0.2	1	0.1	2	0.2
Terminal deletion + terminal duplication involved in one chromosome	5	1.1	3	0.4	8	0.7
Terminal duplication + terminal duplication involved in two chromosomes	6	1.3	0	0.0	6	0.5
Interstitial deletion/duplication	0	0.0	2	0.3	2	0.2
Terminal duplication + interstitial deletion	0	0.0	1	0.1	1	0.1
Microdeletion/microduplication (CNVs < 10 Mb)	**67**	**14.9**	**10**	**1.4**	**77**	**6.6**
Pathogenic CNV	16	3.6	6	0.8	22	1.9
Likely pathogenic CNV	0	0.0	3	0.4	3	0.3
VOUS	51	11.4	1	0.1	52	4.5
UPD	**0**	**0.0**	**8**	**1.1**	**8**	**0.7**
Whole genome UPD	0	0.0	2	0.3	2	0.2
Single-chromosome UPD	0	0.0	1	0.1	1	0.1
Segmental UPD	0	0.0	5	0.7	5	0.4
Total	336	74.8	415	58.4	751	64.7

Significant are in vaue [bold].

*CNV-seq* copy number variation sequencing; *QF-PCR* quantitative fluorescent-polymerase chain reaction; *SNP array* single nucleotide polymorphism array; *UPD* uniparental disomy; *VOUS* variants of unknown significance.

**Figure 3:**
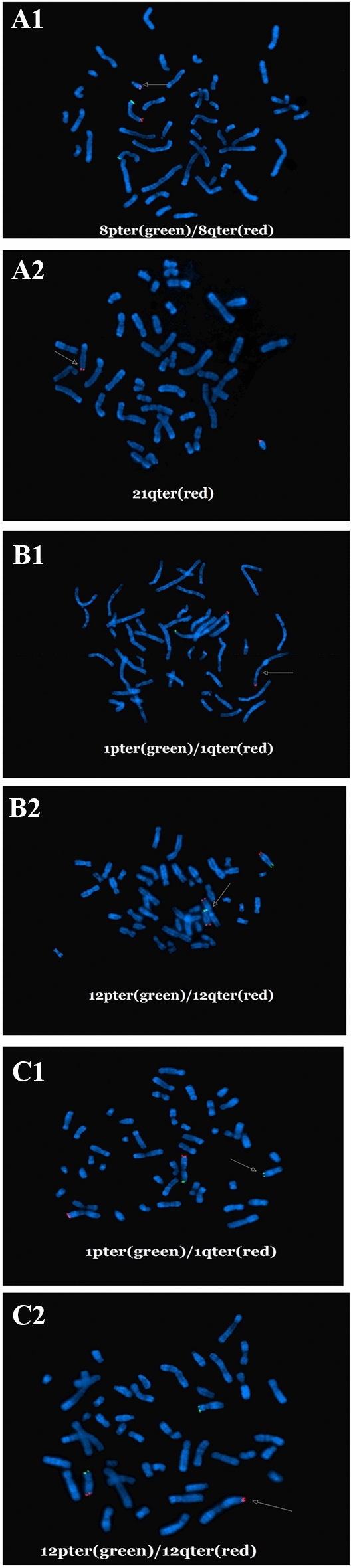
FISH results of case 31 and 54. In case 31, SNP array on abortion villous tissue revealed a 11.8 Mb duplication on 8q24.1q24.3 and a 11.18 Mb deletion on 21q22.12q22.3, whereas parental karyotypes were normal, further FISH analysis result [ish t(8;21)(8p + ,21q + ;8q +)] revealed that copy number variation in aborted embryos is inherited from the father, chart A1 and A2 shows the results of FISH labeling 8p with green signal, 8q with red signal and 21q with red signal, indicating reciprocal translocation with father (arrow); In case 54, SNP array on abortion villous tissue revealed a 15.8 Mb deletion on 1p36.33p36.13 and a 10.8 Mb duplication on 12q24.31q24.33 , whereas parental karyotypes were normal, FISH analysis abortion villous tissue revealed ish der(1)t(1;12)(12q + ,1q +) (B1, B2), further FISH analysis result [ish t(1;12)(12q + ,1q + ;12p + ,1p +)] revealed that copy number variation in aborted embryos is inherited from the mother (C1, C2), chart c and d shows the results of FISH labeling 1p with green signal, 1q with red signal, 12q with red signal, and 12p with green signa indicating reciprocal translocation with father (arrow).

Partial aneuploidy (large CNVs ≥ 10 Mb) was observed in 54 (4.7%) cases (Table 3). Both large deletions and duplications occurred most commonly in chromosome 8, terminal deletion/duplication was identified in 18 (1.6%) cases, accounting for the largest proportion of partial aneuploidies. The distributions of large CNVs and associated genes in CVSs are shown in Table 3. Large copy number losses occurred mostly in chromosome 8, followed by chromosomes X and 7. Large copy number gains also occurred most frequently in chromosome 8, followed by chromosome 9. In addition, a terminal deletion accompanied by terminal duplication involving two chromosomes was detected in 17 cases, suggesting the presence of an unbalanced translocation, which represented the second largest proportion of partial aneuploidies (17 cases; 31.5% of all partial aneuploidies and 1.5% of all analyzed cases), among these, the karyotypes of 16 couples were available, and 14 couples were reported as reciprocal translocation carriers, except for 2 that had normal karyotypes. Samples from the two couples with the normal karyotypes were further subjected to FISH analysis, and two (Case 31 and 54) of them were identified to have submicroscopic reciprocal balanced translocations (see Table 3 and Fig. 3). A terminal deletion coupled with terminal duplication involving one chromosome was detected in 8 cases, suggesting an unbalanced pericentric inversion, which accounted for 14.8% of all partial aneuploidies and 0.7% of all analyzed cases, further parental studies showed 5 of them were derived from inversion) (Table 3), in the remaining 23 cases, parental studies were not available due to their refusal.

**Table 3:** Partial aneuplodies (CNV ≥ 10 Mb) identified in 54 cases of miscarriage.

Case number	Maternal age	Weeks at miscarriage	Molecular karyotyping results	Size (Mb)	Pathogenicity classification	Parental origin	Associated disease (gene)
CNV-seq/QF-PCR							
1	21	10	seq[hg19] dup(7)(p22.3p21.1)chr7:g.43377_19791607dup del(7)(7q31.31q36.3)chr7:118,270,793_159,066,363del	19.7 40.7	P P	mat	
2	23	10^+4^	seq[hg19]del(8)(p23.3p22)chr8:g,160001_13800000del dup(13)(q32.1q34)chr13:g,97820001_115100000dup	13.6 17.3	P VOUS	mat	8p23.1 deletion syndrome
3	27	9 ^+1^	seq[hg19] dup(2)(q37.2q37.3)chr2:g.235953158_243189373dup dup(10)(q23.3126.3)chr10:g.92521431_135524747dup [0.77]	7.2 43	P P	pat	2q37.3 deletion syndrome *ABCC2*, *ACADSB*, *ADD3*
4	27	11	seq[hg19]del(5)p15.33p14.1)chr5:g.10001_28660001del[0.80]/ dup(5)(p14.1p12)chr5:g.28710000-45810000dup[0.20]/ dup(5)(q15q35.3)chr5:g.96660001-180860000dup[0.20]	28.7 17 84.2	P P p		CDCS (*SDHA*, *PDCD6*, *AHRR*) 5p13 duplication syndrome (*LSPIP3*, *CDH6*, *DROSHA*) Partial trisomy5 q syndrome (*RGMB, FLJ35946, CHD1*)
5	31	8	seq[hg19 ]del(22)(q11.1q11.21)chr22:g.16840000_20260000del dup(6)(p22.1q27)chr6:g.28020000_170920000dup	3.42 142.9	P p		DGS (*TBX1*) 6q24 region (*PLAGL1*)
6	25	11^+4^	dup(8q23.3qter)chr8:g. 115,600,000-146300000dup dup(18pterq22.1)chr18:g.20000-61880000Mbdup	30.7 61.9	P p	pat	8q distal trisomy 18p trisomy,18q partial trisomy (*ORPHA*)
7	28	12	seq[hg19]del(2)(q37.1q37.3)chr2:g.232800000_243020000del	10.22	P	mat	2q37 deletion syndrome (*HDAC4*)
8	30	6	seq[hg19]del(7)(q32.3q36.3)chr7:g.130560000_159138663del	28.6	P		LQTS 2; Complex Neurodevelopmental Disorder; holoprosencephaly 3; Currarino syndrome (*KCNH2*、 *KMT2C、SHH* and MNX1)
9	30	12	seq[hg19] del(5)(p15.33p14.3)chr5:g.14160000-15580000del[0.58] dup(5)(p14.1p12)chr5:g.14160000-15580000dup[0.42]	20.6 17.8	P P		CDCS 5p13 duplication syndrome
10	25	10^+3^	seq[hg19] del(8)(p23.3p21.3)chr8:g.160000_22220000del del(8)(p21.2p11.22) (mos)chr8:g.26380000_39480000del	22.1 13.1	P P		8p23.1 deletion syndrome (*GATA4*)
11	34	9	seq[hg19] del(8)(p23.3p22)chr8:g.200001-17800000del dup(8)(q22.2q24.3)chr8:g.9960001-146300000dup	17.6 46.7	P VOUS	pat	8p23.1 deletion syndrome (*GATA4*)
12	36	8	seq[hg19]del(8)(p23.3p21.3)chr8:g.160001-2210000del del(8)(p21.3p21.1)chr8:g.22060001-27860000del	20.4 5.8	P LP		8p23.1 deletion syndrome (*GATA4*) 8p21.2p21.3 deletion syndrome
13	31	12	seq[hg19] dup(14)(q32.1q32.33)chr14:g.94400000-107300000dup	12.9	P		
14	28	12	seq[hg19] del(18)(p11.32p11.21)chr18:g.120000-14980000del	14.9	P		18p deletion syndrome
15	33	8^+4^	seq[hg19] del(7)(q32.2q36.3)chr7:g.129720000-159138663del	29.4	P		*KCNH2,KMT2C,SHH*,and *MNX1*
16	27	9	seq[hg19] del(8)(p23.3p22 )chr8:g.160000-16740000del dup(8)(q13.2q24.3 )chr8:g.70280000-146300000dup	16.6 76.0	P P	pat	8p23.1 deletion syndrome Leri pleonosteosis chromosome duplication syndrome
17	30	9	seq[hg19] del(4)(q34.3q35.2)chr4:g. 179,611,562-190957460del	11.3	LP		
18	33	8^+3^	seq[hg19] del(1)(p36.33p36.13)chr1:g.820000-16240000del dup(14)(q24.3q32.33)chr14:g.77620000-107300000dup	15.4 29.7	P P	mat	1p36 deletion syndrome (*GABRD*)
19	30	9^+1^	seq[hg19] del(1)(q41q44)chr1:g.215716667-2489082del dup(12)(q21.31q24.33)chr12:g.84968124-133841515dup	33.2 48.9		mat	
20	28	9	seq[hg19] del(4)(q32.2q35.2)chr4:g.163350001-191040000del dup(2)(q37.1q37.3)chr2:g.234690001-243090000dup	27.2 8.4		pat	
21	36	8	seq[hg19] del(2)(q37.1q37.3)chr2:g.234816411-243074071del dup(4)(q32.2q35.2)chr4:g.163335055-191044276dup	8.6 27.7	P P	mat	
22	33	7	seq[hg19]dup(13)(q21.33q34)(mos)chr13:g.71700000-115100000dup	43.4	P		
23	28	12	seq[hg19]del(2)(q37.1q37.3)chr2:g.232800000-2430100000del dup(20)(p13p12.2)chr20:g.60000-11100000dup	10.2 11.0	P P	pat	2q37.3 deletion syndrome (*HDAC4*)
24	25	8^+1^	seq[hg19] del(13)(q21.31q34)chr13:g.63100000-115100000del dup(1)(q21.1q44)(mos)chr1:g.144000000-249220000dup	52.0 105.2	P P	pat	Hirschsprung disease (*EDNRB*) holoprosencephaly-5 (*HPE5*) 1q21.1 duplication syndrome (*GJA5*)
25	30	12	seq[hg19] del(8)(p23.3p12)chr8:g.10001-33260001del dup(8)(q13.2q24.3 )chr8:g.70280000-146300000dup	3.3 13.0	P P	pat	Partial monosomy 8p syndrome Trisomy 8q syndrome
26	25	8^+6^	seq[hg19]del(4)(q34.3q35.2)chr4:g.177920001_190940000del dup(9)(p24.3p13.1)chr9:g.200001_38780000 dup	13.0 38.6	LP LP	mat	
27	33	10^+2^	seq[hg19]dup(15)(q22.31q26.3)chr15:g.64480001_102400000 dup del(2)(q37.2q37.3)chr7:g.129720000-159138663del	37.9 6.0	P P	pat	15q26 overgrowth syndrome 2q37 deletion syndrome
28	37	8^+2^	seq[hg19] dup(9)(p24.3p13.1)chr9:g.200001-38780000dup (CN:4)	38.6	P		Tetrasomy 9p (male)
29	29	^11^	seq[hg19]dup(20)(p13p12.2)chr20:g.60000_11100000dup	11.09	P		Familial WPWS
SNP array							
30	33	9	arr[hg19] Xp22.12p11.21(21,782,384–56,905,943) × 1 Xq12q28(65,783,010–155,160,723) × 1	35.1 89.4	P P		*MECP2*、*GRIA3*、*AFF2* and *MAMLD1*
31	30	11^+1^	arr[hg19] 8q24.22q24.3(134,495,771–146,295,771) × 3 21q22.12q22.3 (36,431,283–47,612,400) × 1	11.8 11.3	P P	pat*	
32	29	12	arr[hg19]1q21.1q44(145,744,322–249,224,684) × 2–3	103	P		
33	28	9^+1^	arr[hg19] 8q13.3q24.3(70,949,908–146,295,771) × 2–3 22q13.31q13.33(48,324,664–51,197,766) × 1–2	75.3 2.8	P P	pat	
34	38	11	arr[hg19] 9q22.33q33.3(99,889,390–128,756,159) × 2–3	28.8	P		
35	25	8	arr[hg19] 8p23.3p11.21(158,048–40,469,480) × 1 20p13p12.1(61,661–15,916,956) × 3	40.0 15.8	P P	mat	
36	24	8^**+**1^	arr[hg19] 7q31.2q34(115,729,160–141,679,588) × 3 7q34q36.3(141,687,274–159,119,707) × 1	26 17	P P	mat	
37	30	10	arr[hg19] 5p15.33p14.3(113,576–22,823,694) × 1	22.7	P	dn	CDCS (*CTNND2*)
38	31	9	arr[hg19] 8p23.3p23.1(3,720,080–8,079,920) × 1	11.8	P		8p23.1deletion syndrome (*GATA4*)
39	30	12	arr[hg19] Yp11.31q11.23(2,650,424–28,799,654) × 0–1	26	P		
40	28	8	arr[hg19] 6p25.3q13(294,825–75,334,384) × 3	75	P		Partial 6p trisomy
41	27	9^**+1**^	arr[hg19]4q11q35.2(52,686,030–190,957,460) × 3 4p16.3p16.1(68,345–8,721,580) × 1	138 8.6	P P	pat	Partial 4q trisomy WHS
42	32	10	arr[hg19] 7q34q36.3(142,342,270–159,119,707) × 1, 8q22.3q24.3 (106,063,542–146,295,771) × 3	16.7 40	P P	NA	LQTS (*KCNH2, KMT2C,SHH*) Currarino syndrome( *MNX1*)
43	25	11	arr[hg19] 8p23.3p12(158,048–33,547,773) × 1	33.4	P		8p23.1deletion syndrome (*GATA4*)
44	28	8^+6^	arr[hg19] 5p15.33p15.2(113,576–14,921,416) × 3 11q24.1q25(122,084,943–134,529,443) × 1	14.8 12.4	LP P	pat	CDCS Jacobsen syndrome
45	25	11^+3^	arr[hg19] 4p16.3p15.32(68,345–17,567,960) × 1 6q23.2q27(131,312,783–170,914,297) × 3	17.5 39.6	P P	mat	WHS *PLAG1*, *HYMAI*
46	35	8^+1^	arr[hg19] 8p23.3p12(158,048–35,444,027) × 1	35.0	P		8p23.1deletion syndrome (*GATA4*)
47	26	10	arr[hg19] 15q11.2q25.2(22,770,421–84,834,123) × 3 15q25.2q26.3(85,053,133–102,429,040) × 1	62.1 17.4	P P		PWS/AS *CHD*2, *IGF1R*
48	28	12	arr[hg19] 18q12.1q23(29,715,217–78,013,728) × 3	48.3	P		
49	32	12	arr[hg19] 9p24.3q21.13(208454_77662508) × 3	77.5	P		Partial 9p trisomy
50	33	12	arr[hg19] Xp22.33p11.1(168551_58326434) × 1 Xp11.1q28(58348173_155233098) × 3	58.2 96.9	P P		TS TS
51	26	10	arr[hg19] Xp22.33p22.12(168552_19907735) × 1, Xp22.12q28(20116846_155233098) × 1–2	19.3 135.1	P P		TS TS
52	25	12	arr[hg19] 2p25.3p22.2(12771_37039935) × 3	37	P		
53	29	11	arr[hg19]Xp22.31p11.22(168552_52092488) × 1, Xq13.3q28(75786953_155233098) × 1	51.9 79.4	P P		TS TS
54	35	11	arr[hg19]1p36.33p36.13(849,466–16,724,642) × 1, 12q24.31q24.33(122,915,078–133,777,562) × 3	15.8 10.8	P LP	mat#	1p36 deletion syndrome

*AS* Angelman syndrome ;*mat* maternal; *CDCS* Cri du Chat Syndrome; *CNV-seq* copy number variation sequencing; *CN* copy number; *FISH* fluorescence in situ hybridization; *LQTS* long QT syndrome; *LP* likely pathogenic; *LFU* loss to follow up; *NA* not available; *P* pathogenic; *PWS* Prader-Willi syndrome; *Pat* paternal; *QF-PCR* quantitative fluorescent-polymerase chain reaction; *TS* turner syndrome; *VOUS* variants of uncertain significance; *WHS* Wolf-Hirschhorn syndrome; *WPWS* Wolff-Parkinson-White syndrome.

*In case 31, SNP array on abortion villous tissue revealed a 11.8 Mb duplication on 8q24.1q24.3 and a 11.18 Mb deletion on 21q22.12q22.3, whereas parental karyotypes were normal, further FISH analysis result [ish t(8;21)(8p + ,21q + ;8q +)] revealed that copy number variation in aborted embryos is inherited from the father, chart A1 and A2 shows the results of FISH labeling 8p with green signal, 8q with red signal and 21q with red signal, indicating reciprocal translocation with father (arrow).

^#^In case 54, SNP array on abortion villous tissue revealed a 15.8 Mb deletion on 1p36.33p36.13 and a 10.8 Mb duplication on 12q24.31q24.33, whereas parental karyotypes were normal, FISH analysis abortion villous tissue revealed ish der(1)t(1;12)(12q + ,1q +) (B1, B2), further FISH analysis result [ish t(1;12)(12q + ,1q + ;12p + ,1p +)] revealed that copy number variation in aborted embryos is inherited from the mother (C1, C2), chart c and d shows the results of FISH labeling 1p with green signal, 1q with red signal, 12q with red signal, and 12p with green signal indicating reciprocal translocation with father (arrow).

Submicroscopic CNVs (< 10 Mb) (microdeletions/ microduplications) were identified in 77 (6.6%) cases. The distributions of submicroscopic P/LP CNVs and associated genes in CVSs are shown in Table 4. Among these, 22 (1.9%) were considered pCNVs, and 3 (0.3%) and 52 (4.5%) were classified as likely pCNVs and VOUS, respectively (Table 2).

**Table 4:** Pathogenic/likely pathogenic microdeletions/duplications (CNVs < 10 Mb) identified in 25 cases of miscarriage.

Case ID	Maternal age	Weeks at miscarriage	Molecular karyotyping results	Size (Mb)	Pathogenicity classification	Associated disease (gene)
CNV-seq/QF-PCR						
1	25	12	seq[hg19]del(1)(q21.1q21.1) chr1:g.146520000_147780000del	1.2	P	1q21.1 deletion syndrome (*GJA5*)
2	27	9^+4^	seq[hg19]del(2)(q37.2q37.3) chr2:g.235953158_243189373del	7.2	P	2q37.3 terminal region (*HDAC4*)
3	28	12	seq[hg19]del(17)(q11.2) chr17:g.29061631_30199309) × 1	1.1	P	NF1 microdeletion syndrome (*NF1*)
4	29	10	seq[hg19]del(14)(q13.3q13.3) chr14:g.36725821-37007136del	0.28	P	Brain-thyroid-lung syndrome (*NKX2-1*)
5	30	8 + 4	seq[hg19]del(8)(p23.3) chr8:g.10132-1126452del	1,12	P	Partial monosomy 8p
6	32	10 + 1	seq[hg19]dup(22)(q11.21) chr22:g.18648855_21464764) × 3	2.48	P	22q11.21 microduplication syndrome
7	36	6	seq[hg19]dup(22)(q11.1q11.21) chr22:g.16840000-18640000dup mat	1.8	P	CES (*CECR1*, *CECR2*, *ATP6V1E1*)
8	31	12	seq[hg19]dup(1)(q21.1q21.2) chr1:g.146500000-147400000dup	0.9	P	1q21.1 duplication syndrome (*GJA5*)
9	25	10	seq[hg19]del(22)(q11.21) chr22:g.18630001-20730000del	1.77	P	DGS (*TBX1*)
10	37	12	seq[hg19]del(17)(p12) chr17:g.14160000-15580000del	1.42	P	HNPP (*PMP22*)
11	28	10	seq[hg19]dup(17)(p13.3) chr17:g.1183595–1,424,500	0.38	P	SHFLD3 syndrome (*BHLHA9*)
12	42	9	seq[hg19]del(15)(q11.2) chr15:g.22800001_23100000del	0.30	P	BBS (*NIPA1*, *NIPA2*)
13	28	8	seq[hg19]del(15)(q26.2q26.3)(mos) chr15:g.95520000_100140000del	4.62	P	15q26-qter deletion syndrome (*IGF1R*)
14	29	11^+2^	seq[hg19]del(20)(q13.33) chr20:g.62060000-62220000del	0.16	P	BFNS1 (*KCNQ2*)
15	30	10	seq[hg19]dup (17)(p12) chr17:g.14160000-15580000del	1.42	P	HNPP (*PMP22*)
16	36	8^+3^	seq[hg19]del(X)(q23q24) chrX:g.109340001_119280000del	9.94	P	MRXS14 (*UPF3B*)、MRXSN(*UBE2A*)
SNP array						
17	30	9^+2^	arr[hg19] 10q26.2q26.3 (130,066,717–132,733,665) × 3 10q26.3 (133,858,562–135,426,386) × 1	2.6 1.5	LP LP	
18	27	10	arr[hg19] 5q35.3 (179,249,664–180,715,096) × 1 22q13.2q13.33 (41,167,042–51,099,092) × 3	1.4 9.9	P P	
19	30	11^+1^	arr[hg19] 22q13.31q13.33 (44,261,580–51,197,766) × 1	6.9	P	PMS (*SHANK3*)
20	20	10^+2^	arr[hg19] 15q15.1q15.3 (40,578,808–43,835,278) × 1	3.2	LP	
21	32	8^+6^	arr[hg19] Yp11.31p11.2 (2,650,424–6,356,292) × 0 Yp11.2 (7,251,143–9,745,027) × 0	3.7 2.4	P P	*SRY* *SRY*
22	31	9^+2^	arr[hg19] 1p36.33p36.32 (849,466–2,579,267) × 3 15q26.1q26.3 (94,233,409–102,429,040) × 1	1.7 8.2	LP P	15q26-qter deletion syndrome (*IGF1R*)
23	40	10	arr[hg19] 16p11.2 (29,696,959–30,165,725) × 3	0.47	P	16p11.2 microduplication syndrome
24	34	9^**+1**^	arr[hg19] 17p13.3p13.2 (525–4,931,704) × 1	4.9	P	MDS (*YWHAE* and *PAFAH1B1*)
25	28	8	arr[GRCh37]2p25,3 (1277–2,390,639) × 1	2.4	P	*MYTIL*

*BBS* Burnside-Butler syndrome; *BFNS* benign familial neonatal seizures; *HNPP* hereditary neuropathy with liability to pressure palsies; *LP* likely pathogenic; *MDS* Miller-Dieker syndrome; *PMS* Phelan-McDermid syndrome; *P* pathogenic; *QF-PCR* quantitative fluorescent-polymerase chain reaction; *SHFLD3* split-hand/foot malformation with long bone deficiency 3.

**Figure 4:**
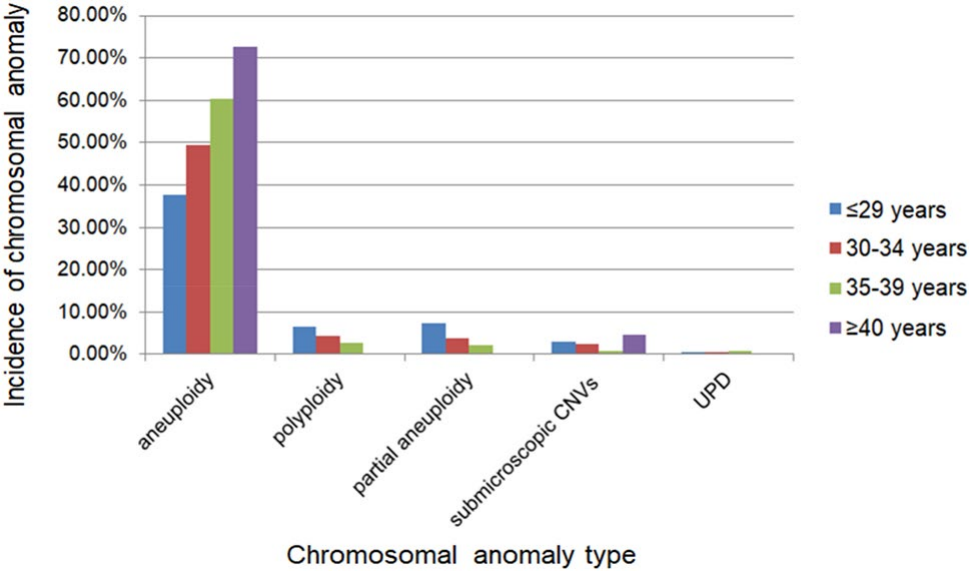
The incidence of chromosomal abnormality for subgroups of different maternal age.

**Figure 5:**
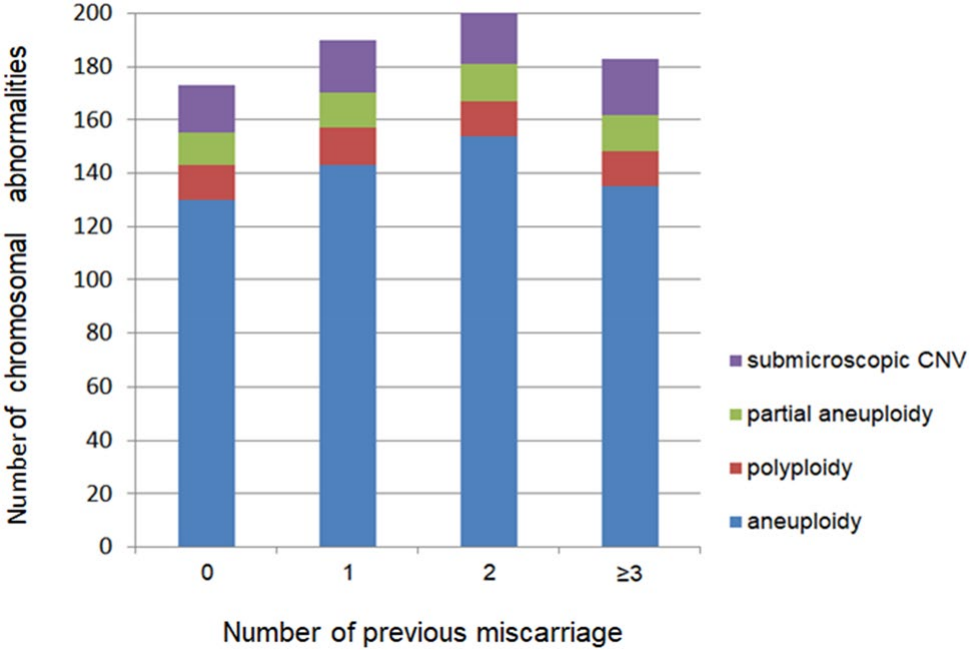
The number of chromosomal abnormality in subgroups of previous miscarriage.

Thirty-nine and twenty-nine cases with pCNVs were found in ≤ 29-year-old and 30–34-year-old pregnant women, respectively. There were only 7 and 2 pCNVs in CVSs detected in the 35–39 and ≥ 40-year-old age groups (Fig. 4). The number of chromosomal variations in the subgroups with previous miscarriages is shown in Fig. 5. There were no significant differences in the different types of chromosomal abnormalities among the four groups (*p* > 0.05).

In addition, we identified 11 pCNVs (9 deletions and 2 duplications) which were detected in CVSs at least twice. These pCNVs involved deletions in 1q21.1, 8p23.1, 2q37.3, 5p15.33p14.3, 7q32.3q36.3, 17p12, 15q26.2q26.3, 4p16.3p16.1, and Xp22.3q28, as well as duplications in 5p13 and 8q23.3q24.3 with sizes ranging from 7.2 to 96.9 Mb.

UPD was detected in 8 (0.7%) cases, who all denied any known history of parental consanguinity. Among these, whole-genome UPD, suggestive of molar pregnancy, was observed in 2 cases. Single-chromosome UPD (involving chromosome 13) was identified in one case. In addition, five cases of segmental UPD (involving chromosomes 3p, 5p, 6p, 7q, 8q, 9p, 9q, 11q, 14q, 16q and 22q) were observed.

### The associations between chromosomal abnormalities of CVSs and maternal age

We investigated the relationship between maternal age and chromosomal abnormalities, finding that the frequency of aneuploidy increased with maternal age. The incidence of chromosomal aneuploidy abnormalities among women aged ≤ 29, 30–34, and 35–39 years was all significantly lower than that in women ≥ 40 years old (*p* < 0.001), whereas the frequencies of other types of chromosomal abnormalities were not correlated with maternal age (*p* > 0.05). The correlation between chromosomal abnormalities and maternal age is presented in Fig. 4.

## Discussion

Early pregnancy loss puts a heavy psychological burden on most couples. Embryonic numerical and structural chromosomal abnormalities are the most common cause of early pregnancy loss^6,19^. Chromosomal aberration analysis of CVSs is essential for assessing the causes of first-trimester miscarriages. In the current study, we aimed to evaluate the incidence and distribution of chromosomal abnormalities detected by CMA and CNV-seq/QF-PCR in CVSs samples.

Until now, karyotyping, CMA, as well as CNV-seq were the main technologies for identifying chromosomal anomalies^19–21^. Karyotyping is considered the “gold standard” for cytogenetics, its advantage is that it can detect chromosomal numerical, and visible structural abnormalities. However, this technique requires cell culture. Furthermore, its resolution is too low to detect submicroscopic CNVs. Thus, the total detection failure rate is ~ 20%^22^, which sometimes yields false-negative results due to the excessive growth of maternal cells relative to chorionic villus cells. CMA is a first-tier method in detecting CNVs in spontaneous miscarriages^23^. Although SNP array has significant advantages in detecting non-equilibrium chromosomal abnormalities, triploidy, and UPD, it has a relatively low throughput and a high detection cost. Recently, CNV-seq is gradually being used in detecting the genetic causes of CVSs^24^. Both molecular karyotyping technologies do not require cell culture and are reliable in detecting CNVs^25^. Compared with CMA, CNV-seq/QF-PCR has a higher throughput, a more accurate CNV breakpoint detection with 1 × depth, low cost, and a short reporting period, and has a lower level (≥ 10%) of mosaicism and polyploidy detection. We summarized the total detection efficiencies of SNP array and CNV-seq technologies on CVSs. In this study, all 449 and 711 CVSs were successfully examined via CNV-seq/QF-PCR and SNP array, respectively, thus, the detection success rate of both methods was 100%. More normal female samples were observed than normal male samples (213 vs. 196), which was likely caused by confusion of 69 XXX and 46 XX, which is significantly higher than the mean ratio of 0.71 for cases investigated in multiple large-scale products-of-conception studies using cytogenetic analysis^26^.

The overall rate of clinically significant chromosomal abnormalities was 59.7% (693/1160) (Table 2), which is similar to the reported rates in previous researches^27^. Chromosomal numerical anomaly is the most common genetic cause of spontaneous miscarriages^28^. Here, 615 cases (81.9%, 615/751) had numerical abnormalities, which is consistent with results from a recent report^9^, including 531 cases (45.8%) of single chromosome aneuploidy and 31 (2.7%) cases of multiple aneuploidies (Table 2). The occurrence frequency of each chromosome aneuploidy was analyzed in all 531 cases in this study (Fig. 2), which showed that non-segregation errors occurred in all chromosomes except chromosome 1, which is consistent with a previous study^29^.

As the longest human chromosome, trisomy 1 appears to be a very early lethal anomaly that may affect early embryonic development, causing pregnancy failure or biochemical pregnancy^30^. As shown in Fig. 2, trisomy 16 accounts for 81% (430/531) of autosomal trisomy cases, which is in line with the previous report^31^. Among the cases of single autosomal trisomy, the incidence of trisomy 16 was the highest (18.7%, 115/615), followed by trisomy 22 and trisomy 21 (Fig. 2), which is consistent with the reported research^6^. Non-separation of homologous chromosomes causes aneuploidies during the meiotic divisions of germ cells^32^. The development and survival of zygotes with trisomies are inhibited, resulting in early pregnancy loss.

Triploidy is a common chromosomal aberration that usually results in early miscarriage^33^. In our study, polyploidy was found in 50 (4.3%) cases: triploidy in 45 (3.9%) cases, and hypotriploidy in 5 (0.4%) cases, which are slightly lower than those reported by Wang Y.^6^ This difference may be due to inadequate sample size in our study.

Monosomies are lethal due to their gene dose effect; thus, embryonic monosomies often occur very early in pregnancy and lead to spontaneous abortions^34^. Here, monosomies were identified in 19.0% (101/531) of the cases with single aneuploidy, of which, monosomy X accounted for 91.1% (92/101). Unlike autosomal trisomy, the risk of monosomy X did not increase with maternal age, consistent with previous reports^3^. Hassold et al.^35^ found that the paternal loss of a sex chromosome was the most common reason leading to 45, X, which was likely a result of meiotic errors.

The third-child policy in China led to an increasing proportion of AMA. The risk of chromosomal aneuploidies in aborted embryos increases with maternal age^36^. This may be due to egg aging which can cause meiosis without dissociation^36^. Our data also support this point.

Generally, large CNVs which contain numerous genes are expected to be lethal and are known to cause miscarriage^6^. In this study, 79 large CNVs (≥ 10 Mb) in 54 cases were identified. Among 54 cases of large CNVs, thirty were unbalanced rearrangements, with 21 of them confirmed to be of parental origin (16 cases of translocations and 5 cases of inversion) in the subsequent conventional karyotyping/FISH of couples. In addition, we also found that both large deletions and duplications occurred most commonly in chromosome 8, which is consistent with the recent report^6,37^. One likely explanation is that maternal 8p inversion, which is delimited by the olfactory receptor (OR) gene clusters, and may confer susceptibility to unequal crossovers between two OR gene clusters^38^. The number of previous miscarriages ranged from 0 to 4. Approximately 2.7–6.7% of chromosomal balanced rearrangement carriers have experienced recurrent spontaneous abortion (RSA), which indicates that chromosomal balanced rearrangement is one of the main causes of RSA. Our data indicate that the genetic cause of miscarriage should be conducted routinely, abnormal CVSs results can suggest balanced abnormalities in parents, and follow‐up parental testing allows for further refining recurrence risk and whether or not prenatal and/or pre-implantation genetic testing (PGT) needs to be conducted^39^. With exact chromosomal diagnosis, PGT was recommended to these couples^40^. We emphasized that an obstetrician should be aware of submicroscopic reciprocal translocation rearrangement in couples with RSA.

It is worth pointing out that in 2 couples with normal karyotypes, subsequent peripheral blood FISH was performed to confirm whether there is occult chromosomal rearrangement; both cases were proven to be hereditary from the parental submicroscopic reciprocal balanced translocations (Cases 30 and 53). Thus, submicroscopic reciprocal translocation, even in embryotic large pCNVs in couples with RSA, were sometimes ignored. Further FISH confirmation is recommended for carriers with suspected occult translocations or similar terminal chromosomal banding involving two chromosomes.

However, the role of submicroscopic CNVs in early pregnancy loss is unclear. Specific information based on large cohorts regarding the association between submicroscopic CNVs and miscarriage is limited. Nevertheless, recent studies have shown that dosage changes or mutations of genes that play an important role in early embryonic development could also cause miscarriage^41^. A total of 77 (6.6%) cases of submicroscopic (< 10 Mb) CNVs were identified in the study, similar to those in Levy B’s research^37^. Up to now, little is known about the association between a specific submicroscopic pCNV and miscarriage.

We identified submicroscopic pCNVs in 2.2% (25/1160) of our cases, which is same to 2.2% by Wang et al.^6^. Three recurrent pCNVs, including 22q11.2 microdeletion, 7q11.23 microdeletion, and 16p13.11 microduplication, were reported in previous studies on miscarriage^21,37,42,43^. The possible underlying mechanism that results in early embryonic death may be a malformed fetal cardiovascular system resulting from 22q11.2 deletion and 7q11.23 deletion^6^. Whereas, whether 16p13.11 microduplication contributes to miscarriage remains to be uncertain. In addition, microdeletions in 22q11.21, 2q37.3 and 9p24.3p24.2 as well as CNVs in 11p15.5 were identified as likely to be associated with miscarriage^44^, the potential mechanism is that aberrant methylation or duplication of imprinted genes in 11p15.5 could cause miscarriage. Likewise, Nagirnaja et al.^45^ identified CNV in 5p13.3, interrupting the PDZD2 and GOLPH3 genes, and showed that which was significant correlated with an increased risk of spontaneous abortion (SA). Recently, Sheng et al.^46^ also identified two recurrent RSA-associated CNVs (duplications at 16q24.3 and 16p13.3) compared with the SA.

In our study, we also identified microdeletion in 2q37.3 and 22q11.2 which may lead to miscarriage. In addition, eleven pCNVs, which were detected in CVSs at least twice, involved deletions at 1q21.1, 8p23.1, 2q37.3, 5p15.33p14.3, 7q32.3q36.3, 17p12, 15q26.2q26.3, 4p16.3p16.1, and Xp22.3q28, as well as duplications in 5p13 and 8q23.3q24.3. The association of miscarriage with other submicroscopic pCNVs identified in our cohort, is still unclear, and more large-scale studies are needed to confirm whether these submicroscopic pCNVs contribute to pregnancy loss. Recently, whole-exome sequencing has been applied to study RSA and a few embryonic lethal genes have been discovered^47^. We believe future large-scale cohort will identify more recurrent pCNVs and gene mutations concerning pregnancy loss.

Both SNP array and CNV-seq/QF-PCR were effective in identifying chromosomal aneuploidies, CNVs and polyploidies^48^. However, CNV-seq/QF-PCR and SNP array cannot detect all polyploidies, such as 69, XXX and tetraploidy, as well as balanced structural rearrangement. In addition, CNV-seq can detect mosaicism in at least 10% of CNVs, while microarray can detect mosaicism in at least 30% of CNVs and UPD.

Unlike embryonic chromosomal aneuploidies, the risk of pCNVs in aborted embryos is independent of maternal age^49^. Our data indicate that the incidence of other chromosomal abnormalities such as polyploidy, CNVs, and UPD was not directly correlated with maternal age (*p* > 0.05), which is partially consistent with the study reported by Larroya et al^50^. Based on this point, SNP array or CNV-seq/QF-PCR analysis on CVSs is strongly recommended, regardless of maternal age.

In the present study, the chromosomal structural abnormality rate (6.8%, 18/264) of CVSs samples in AMA was lower than that (12.6%, 113/896) of miscarriage samples in YMA, which is in line with a previous research^51^, because the frequency of embryotic chromosomal structural abnormalities did not seem to be correlated with AMA. A larger population study is needed to further clarify the noncorrelation.

UPD is a rare chromosomal abnormality. The major mechanism of UPD formation is trisomy rescue^52^. Specific regions on chromosomes 6, 7, 11, 14, 15, and 20 can result in genomic imprinting diseases. Chromosome-wide UPD resulted from parthenogenesis, which is one of the genetic factors for early pregnancy loss^53^. The incidence (0.7%) of UPD in our study might have been underestimated since UPDs were not detected in the cases via CNV-seq/QF-PCR. Single-chromosome UPD is presumably suggestive of a monosomy rescue event. Segmental UPD in these cases would likely be due to a meiotic/mitotic error, in which a meiotic non-disjunction event was followed by a mitotic cross-over between the paternal and maternal homologs, with subsequent trisomy rescue^54^. Pregnancy loss could be due to UPD resulting in unmasking of an underlying lethal recessive disease gene(s) or imprinted genes.

The limitation of this study is that the number of cases is not large enough; the limited data is not fully representative of the CNV characteristics of early miscarriage. Thus, the correlations among the history of previous abortions/weeks of miscarriage, modes of conception, and embryonic chromosomal abnormalities have not yet been evaluated. For this, a larger population study and enrichment analysis of genetic functions within the examined CNVs will be necessary to identify the specific genes associated with miscarriage.

In summary, our study suggests both SNP array and CNV-seq/QF-PCR are reliable, robust, and high-resolution technologies for genetic diagnosis of miscarriage, especially in detecting CNVs.

## Data Availability

The data that support the findings of this study are available from the corresponding author upon reasonable request.

## Abbreviations

CNV: Copy number variation
CMA: Chromosomal microarray analysis
CVSs: Chorionic villus samples
FISH: Fluorescence in situ hybridization
NGS: Next generation sequencing
SNP: Single nucleotide polymorphism
VOUS: Variant of uncertain significance
UPD: Uniparental disomy
